# Extracellular Chromatin Traps Interconnect Cell Biology, Microbiology, and Immunology

**DOI:** 10.3389/fimmu.2013.00160

**Published:** 2013-06-24

**Authors:** Marko Radic, Mariana J. Kaplan

**Affiliations:** ^1^Department of Microbiology, Immunology and Biochemistry, University of Tennessee Health Science CenterMemphis, TN, USA; ^2^Division of Rheumatology, Department of Internal Medicine, University of Michigan Medical SchoolAnn Arbor, MI, USA

In a scientist's career, the often elusive goal is to discover a biological process that is both unexpected and, once revealed, of obvious importance. In that case, the new observation will attract the attention of others and prove to be of crucial significance in several overlapping areas of science. Nearly a decade after the original discovery of neutrophil extracellular traps (NETs) by Brinkmann et al. (2004), NETs have been firmly established as a fundamental biological mechanism used by neutrophils to respond to infections. Scores of additional reports confirmed and extended the initial observations to provide insights into the contributions of NETs to bacterial, fungal, viral, and protozoan infections (Brinkmann and Zychlinsky, 2012). NET release was shown to be the regulated outcome of a programed cell death process designated NETosis, and NETs were linked to autoimmunity and cardiovascular disease. As Editors, we are pleased by the enthusiastic response of 20 groups of scientists from 4 continents who participated in our effort to summarize, evaluate, and extend understanding of NETosis. This booklet captures some of the excitement that was shared with us by the authors.

Goldmann and Medina (2013) introduce the topic of NETosis by describing aspects that differentiate NETosis from other forms of cell death and by reminding the readers that extracellular traps are produced by eosinophils, mast cells, and even monocytes/macrophages in addition to neutrophils. Thus, NETosis is more accurately called ETosis. In alternative manner, DNA for ETs can be derived from the nucleus or from mitochondria and the DNA can be released without completely inactivating all functions of the cell releasing the DNA. The authors provide a careful summary of steps in ETosis and illustrate the process with electron micrographs, while reminding us that many open questions still remain.

Brinkmann et al. (2013) present a semi-automated method for enumerating cells that traverse sequential stages in NETosis. This useful and easily adaptable method by the original discoverers of NETs relies on dual channel fluorescence and compares binding of anti-chromatin antibodies relative to staining with a DNA-intercalating dye. Image analysis computes the percentage of NETting neutrophils. The authors give examples of Toll-like receptor stimuli, crystals, and cytokines that induce NETosis. The new method should be ideally suited to high throughput screening for drugs that affect the efficiency of NET release.

Additional contributors discuss mechanistic features of NETosis. Rohrbach et al. (2012) focus on the role of peptidylarginine deiminase 4 (PAD4) in the regulation of NETosis. By reviewing PAD4 structure and function, the authors discuss inhibitors of PAD4 and their potential use in suppressing NETosis. PAD4 is a particularly appealing target for inhibition (or enhancement) of NETosis because the reaction pathway of this enzyme is understood in molecular detail.

Leshner et al. (2012) provide an elegant illustration of PAD4’s potential to induce NETs. They demonstrate that overexpression of PAD4 in heterologous cells leads to chromatin decondensation and unfolding of NET-like chromatin. Induced PAD4 expression leads to histone deimination and the release of NET-like webs from U2OS and NIH 3T3 cells. Specifically, the authors show that deimination of arginines in the amino terminus of histone H3 reduces the binding of the heterochromatin protein HP1β to the adjacent lysine 9 in H3. The disruption of higher order chromatin packing may provide a molecular switch that regulates NET release in NETosis.

Neeli and Radic (2013) observed that two ancient regulatory enzymes from the protein kinase C (PKC) family exhibit opposite effects on PAD4 activity. The authors used PMA and ionophore, two compounds that induce NETosis, along with inhibitors of PKC activity, to identify PKCα as an inhibitor of PAD4 and PKCζ as a facilitator of PAD4-mediated histone deimination. The authors conclude that evolutionary pressure ensured precise regulation of histone deimination because NETs make important, yet potentially dangerous contributions to innate immunity.

The important question of whether extracellular chromatin traps are involved in autoimmune disease is critically reviewed by two groups of scientists. Darrah and Andrade (2013) present an analytical comparison of different modes of cell death and contrast the potential contributions of apoptosis, necrosis, and NETosis to the release of nuclear autoantigens. The authors survey different possibilities that may link NETosis to the stimulation of the adaptive immune system in systemic autoimmune disorders. The large number of autoantigens that are integral components of NETs provide a compelling argument for NETosis’ role in the pathogenesis of autoimmune disorders and the loss of immune tolerance.

Knight et al. (2012) take a broad view to summarize evidence linking NETosis with specific aspects of autoimmune disorders. By highlighting recent findings from studies of small vessel vasculitis, psoriasis, gout, Felty's syndrome, and systemic lupus, the authors suggest two mechanisms for how NETs could participate in disease pathogenesis. One mechanism involves the contribution of NETs to organ damage; the other sees NET proteins as the trigger or exacerbating factor in autoimmunity. In particular, post-translational modifications of NET proteins are highlighted as features that make them into “altered self,” autoantigens that may break tolerance and initiate autoimmunity.

Schorn et al. (2012) investigate sterile inflammation that is caused by crystals that form in the joints and kidneys of patients suffering from gout. The crystals consist of monosodium urate (MSU) and induce NETosis in purified neutrophils. The NETosis is dependent on reactive oxygen production, thus it likely follows established NETosis pathways. The authors detect NETs in biopsies of gouty arthritis patients and examine downstream pathways involved in clearance of NETs. Surprisingly, they find that molecules that function in the clearance of apoptotic cells (complement factor C3b, C-reactive protein, and galectin-9) have limited affinity for NET chromatin and thus may not effectively contribute to NET clearance.

Kambas et al. (2012) review evidence implicating neutrophils and NETs in the release of tissue factor (TF), an important regulatory protein that orchestrates initial steps in coagulation and whose excess may directly lead to thrombosis. Even though neutrophils are clearly involved in thrombotic events, the issue of neutrophil production of TF is more controversial. The authors review recent publications in this area and highlight their own data that identify TF as a component of NETs with important implications for thrombosis.

Hahn et al. (2012) summarize evidence for the participation of NETs in reproductive complications, ranging from infertility to preeclampsia and fetal loss. The authors introduce this fascinating topic by reviewing the essential role of neutrophils in the estrus cycle and pregnancy. They shift focus to data suggesting that neutrophils are involved in the complications of preeclampsia, the sudden rise in blood pressure that can jeopardize the life of the mother and fetus. The proposed role of NETosis is supported by an activated, pro-inflammatory state of neutrophils in normal pregnancy, high levels of neutrophil elastase (a NET component) in preeclampsia, and detection of cell-free DNA in circulation. The authors describe reactions between normal neutrophils and highly purified placental micro-debris to argue for the involvement of NETs in preeclampsia.

Parker and Winterbourn (2013) highlight contributions of reactive oxygen products and specific enzymes such as myeloperoxidase (MPO) in facilitating NET release. These authors point to the association of MPO and neutrophil proteases with NET chromatin to connect reactive oxygen functions in NETosis and in bacterial killing. Two major insights arise from these studies. First, different stimuli induce NET release by different mechanisms. Thus, NETosis is MPO-dependent if PMA is the stimulus, whereas MPO is dispensable if *Pseudomonas aeruginosa* induces NETs. Second, bacterial killing by NETs is enhanced if exogenous H_2_O_2_ is supplied, suggesting that MPO in NETs converts H_2_O_2_ to more potent bactericidal oxygen species.

Almyroudis et al. (2013) focus on NADPH oxidase contributions to NETosis by highlighting the different effects of NETosis and apoptosis on immune responses and inflammation. The authors argue that for inflammation to be turned off following clearance of an infectious threat, the form of cell death needs to switch from NETosis to apoptosis. The authors review genetic factors that regulate NADPH and NETosis to conclude that both processes provide opportunities for the development of therapeutics.

Hosseinzadeh et al. (2012) present new data on the development and testing of tempol, a compound that has promise as an effective and relatively safe inhibitor of NETosis. Tempol is a low molecular weight compound that easily passes the plasma membrane and acts as a scavenger of reactive oxygen. The authors test tempol by using a real-time fluorescence assay during neutrophil phagocytosis, chemotaxis, and NETosis. Tempol inhibits NETosis induced by PMA or *Candida albicans*, suggesting its potential use in clinical conditions with excessive NET release.

Lu et al. (2012) review NETosis in infectious diseases and stress that much more needs to be learned about the precise benefits of NETosis in bacterial infections. More specifically, the authors remind us that certain bacterial pathogens such as *Staphylococcus aureus* have a plethora of virulence factors that exploit weaknesses in the immune defenses of the host. Possibly, bacteria may take advantage of neutrophil mechanisms that lead to NETosis. The authors further argue that phagocytosis is the most effective way to dispose of infectious bacteria because fusion of phagosomes and granules produces an environment with highly concentrated bactericidal compounds. In contrast, NETosis diffuses these compounds and is thus less efficient. In that light, pathogens such as staphylococci benefit from the lysis of neutrophils and can also escape from NETs by means of a bacterial nuclease. Thus, NETosis may offer only limited benefits in certain infections but may still enhance collateral damage in the host.

Cheng and Palaniyar (2013) examine NETosis in the context of lung infection and inflammation. Infections that induce NETosis in the lungs are contrasted with inflammatory disorders that lead to tissue injury. Among the lung diseases with suspected NET involvement are acute lung injury and acute respiratory distress syndrome. NETs also may be the potential culprits in the development of cystic fibrosis and asthma, thus making the development of therapies for suppressing NETosis a priority.

Daigo and Hamakubo (2012) present a detailed look at the interaction between pentraxin 3, a soluble pattern recognition receptor, and NETs. Because pentraxin 3 was identified as a component of NETs, the authors purify pentraxin 3 complexes from sepsis patients and carry out their proteomic analysis. They discover that several NET components associate with pentraxin 3 and conclude that pentraxin 3 complexes capture circulating pathogens and deliver them for clearance to phagocytes. The detailed analysis of pentraxin 3 complexes should yield new markers for infectious and inflammatory diseases.

Narayana Moorthy et al. (2013) raise the important question whether NETs are protective or detrimental in secondary bacterial infections. Secondary pneumococcal infections are the major cause of serious complications following seasonal flu infections. The authors quantitate the increased severity of infections in mice by using a grading system for NET release in the lungs. Of relevance, the authors report that *Streptococcus pneumoniae* express a nuclease that makes them impervious to the bactericidal effects of NETs and thus account for why NETs are not more effective in suppressing the bacterial infections.

Abi Abdallah and Denkers (2012) summarize recent data indicating that NETs participate in immune responses to protozoan parasites. More specifically, reports link infections by *Toxoplasma gondii*, *Plasmodium falciparum*, *Eimeria bovis*, and *Leishmania parasites* to the production of NETs. In some cases, NETs may limit the mobility and infectivity of the parasites and lead to the killing of the pathogen. However, at least some protozoan species may have evolved unique mechanisms for escaping neutrophil traps. In addition, protozoan infections may contribute to manifestations of autoimmunity by modifying NET components.

Berger-Achituv et al. (2013) report exciting new findings from a small group of Ewing sarcoma patients in whom tumor-infiltrating neutrophils were detected and analyzed for induction of NETosis. Preliminary data indicate that the release of NETs within or adjacent to the tumors has a negative effect on the outcome of cancer therapy and disease relapse. The topic of tumor-associated neutrophils and inflammation is rapidly gaining momentum in the cell biology of cancer. The discovery of NETs in the vicinity of tumors and their potential role in tumor progression and metastasis will certainly attract growing attention in the near future.

Mohanan et al. (2013) carry out experiments that link obesity with macrophage release of extracellular trap-like chromatin in adipose tissue. Because the death and clearance of adipocytes is implicated in the inflammatory cytokine release by fat tissue macrophages, the authors predicted that macrophage may release extracellular traps upon entering areas of high adipocyte turnover. The results indicate that macrophages within adipose tissues express PAD2 and some of them contain citrullinated histone H4. These data will inspire future studies to determine the role of macrophage traps in adipose tissue.

Nakazawa et al. (2012) complete our collection of articles with a clinical case study that analyzes the cause of death in a patient experiencing microscopic polyangiitis and deep vein thrombosis. In this type of vasculitis, thrombotic complications are common, and the authors expand on previously published data linking NETs to disease severity and progression.
